# Enhancement of ventricular-subventricular zone-derived neurogenesis and oligodendrogenesis by erythropoietin and its derivatives

**DOI:** 10.3389/fncel.2013.00235

**Published:** 2013-11-27

**Authors:** Naoko Kaneko, Eisuke Kako, Kazunobu Sawamoto

**Affiliations:** ^1^Department of Developmental and Regenerative Biology, Nagoya City University Graduate School of Medical SciencesNagoya, Japan; ^2^Department of Anesthesiology and Medical Crisis Management, Nagoya City University Graduate School of Medical SciencesNagoya, Japan

**Keywords:** ventricular-subventricular zone, neurogenesis, oligodendrogenesis, erythropoietin, regeneration, differentiation, neural stem cells

## Abstract

In the postnatal mammalian brain, stem cells in the ventricular-subventricular zone (V-SVZ) continuously generate neuronal and glial cells throughout life. Genetic labeling of cells of specific lineages have demonstrated that the V-SVZ is an important source of the neuroblasts and/or oligodendrocyte progenitor cells (OPCs) that migrate toward injured brain areas in response to several types of insult, including ischemia and demyelinating diseases. However, this spontaneous regeneration is insufficient for complete structural and functional restoration of the injured brain, so interventions to enhance these processes are sought for clinical applications. Erythropoietin (EPO), a clinically applied erythropoietic factor, is reported to have cytoprotective effects in various kinds of insult in the central nervous system. Moreover, recent studies suggest that EPO promotes the V-SVZ-derived neurogenesis and oligodendrogenesis. EPO increases the proliferation of progenitors in the V-SVZ and/or the migration and differentiation of their progenies in and around injured areas, depending on the dosage, timing, and duration of treatment, as well as the type of animal model used. On the other hand, EPO has undesirable side effects, including thrombotic complications. We recently demonstrated that a 2-week treatment with the EPO derivative asialo-EPO promotes the differentiation of V-SVZ-derived OPCs into myelin-forming mature oligodendrocytes in the injured white matter of neonatal mice without causing erythropoiesis. Here we present an overview of the multifaceted effects of EPO and its derivatives in the V-SVZ and discuss the possible applications of these molecules in regenerative medicine.

## Production of neuronal and oligodendrocyte progenitors in the V-SVZ

### New neuron production in the V-SVZ

Neural stem cells (NSCs) in the ventricular-subventricular zone (V-SVZ), located at the lateral walls of the lateral ventricles, have been investigated as an endogenous cell source for neurons (Belvindrah et al., 2009; Kriegstein and Alvarez-Buylla, 2009; Ihrie and Alvarez-Buylla, 2011; Ming and Song, 2011) and oligodendrocytes (Nait-Oumesmar et al., 2008; Gonzalez-Perez and Alvarez-Buylla, 2011) in the postnatal brain. While maintaining themselves by self-renewing cell division, the NSCs produce actively proliferating intermediate progenitors called transit-amplifying cells, which generate immature new neurons, called neuroblasts. The neuroblasts born in the V-SVZ are characterized by their prominent migration capacity. They have a bipolar shape with leading and trailing processes, and in the rostral migratory stream, they migrate in chain-like aggregates for a long distance to the olfactory bulb, where they differentiate into interneurons to be integrated into the olfactory circuitry (Luskin, 1993; Lois and Alvarez-Buylla, 1994; Petreanu and Alvarez-Buylla, 2002; Carleton et al., 2003).

These neurogenic cells are tightly associated with the vasculature. NSCs extend their process with an endfoot that makes contact with blood vessels (Mirzadeh et al., 2008; Tavazoie et al., 2008), the transit-amplifying progenitors reside very close to vessels (Shen et al., 2008; Tavazoie et al., 2008; Kokovay et al., 2010), and the neuroblasts frequently migrate along vessels (Snapyan et al., 2009; Whitman et al., 2009). Therefore, although its precise function remains unclear, the vasculature is thought to contribute to the neurogenic function of the progenitor cells and the efficient migration of neuroblasts (Ihrie and Alvarez-Buylla, 2011).

### Oligodendrocyte production in the V-SVZ

Oligodendrocytes form the myelin sheath, which wraps around axons to facilitate the rapid, saltatory conduction of electrical impulses along them. In early postnatal stages, V-SVZ is an important source of forebrain oligodendrocyte progenitor cells (OPCs) (Levison and Goldman, 1993; Luskin, 1993; Ivanova et al., 2003; Suzuki and Goldman, 2003). These OPCs differentiate into mature oligodendrocytes after they migrate to and colonize the parenchyma. However, a portion of the OPCs remain as progenitors, which are called NG2 glia due to their expression of the OPC marker NG2 proteoglycan, and become a major provider of oligodendrocytes in later postnatal stages and throughout adulthood. (Stallcup and Beasley, 1987; Gensert and Goldman, 1997; Nishiyama et al., 2002; Greenwood and Butt, 2003; Fancy et al., 2004).

Recent studies revealed that the adult V-SVZ continuously produces OPCs, via a distinct group of transit-amplifying progenitors that express the oligodendrocyte lineage markers Olig2 and NG2 (Levison and Goldman, 1993; Nait-Oumesmar et al., 1999; Picard-Riera et al., 2002; Hack et al., 2005; Menn et al., 2006). Although the adult V-SVZ-derived OPCs are a minor population among the entire OPC pool in the brain, they have a distinctive capacity to migrate actively over long distances into the corpus callosum, striatum, and fimbria fornix, where they differentiate into mature, myelin-forming oligodendrocytes (Nait-Oumesmar et al., 1999; Menn et al., 2006; Aguirre et al., 2007). Like the migrating neuroblasts, the migrating OPCs have a bipolar morphology with leading and trailing processes, however, they tend to migrate individually rather than by forming chain-like aggregates (Menn et al., 2006).

### Neurogenesis and oligodendrogenesis under pathological conditions

Cell proliferation in the V-SVZ is up-regulated in response to various pathological conditions that cause neuronal loss, such as ischemic stroke and neurodegenerative diseases. The neuroblasts generated in the V-SVZ migrate toward the injured area and differentiate into functional mature neurons (Yamashita et al., 2006; Kojima et al., 2010; Yoshikawa et al., 2010).

Myelin degeneration blocks conduction, whether it occurs in the context of oligodendrocyte-specific disorders, such as multiple sclerosis, or as a result of non-specific insults, including severe ischemia. The blocked conduction induces a variety of neurological impairments. After demyelination, new oligodendrocytes generated from the parenchymal OPCs contribute to remyelination (Reynolds et al., 2002; Aguirre et al., 2007; Fancy et al., 2009, 2011; Patel et al., 2010; Azim and Butt, 2011; Huang et al., 2011; Mi et al., 2011). In addition, in the brains of multiple sclerosis patients and of rodent demyelination models, new OPC production in the V-SVZ is significantly increased, and these OPCs also contribute to remyelination (Nait-Oumesmar et al., 1999, 2007). Furthermore, V-SVZ-derived migrating progenitors committed to the neuronal lineage can apparently change their fate to differentiate into oligodendrocytes in demyelinated areas (Jablonska et al., 2010).

The insult-induced spontaneous regeneration of neurons and oligodendrocytes is insufficient for full structural and functional restoration of the injured brain. Therefore, interventions to enhance these processes are being sought for future clinical applications. Various interventions that increase new neurons/oligodendrocytes have already been shown to improve neurological function (Leker et al., 2009; Lindvall and Kokaia, 2010; Nakaguchi et al., 2011; Christie and Turnley, 2012). Erythropoietin (EPO), a clinically used erythropoietic factor, is one of the promising candidate drugs that promote V-SVZ-derived neurogenesis and oligodendrogenesis.

## Endogenous EPO activity in the central nervous system (CNS)

### EPO and its receptor

EPO is a single polypeptide glycoprotein hormone that is mainly produced in and secreted from fetal hepatocytes and interstitial fibroblasts in the adult kidney, to increase the number of circulating erythrocytes (Marti, 2004). It consists of 166 amino acids folded into 4 α-helices, and includes three *N*-glycosylation sites that each accommodate up to four sialic acid residues (Jacobs et al., 1985; Lin et al., 1985; Lai et al., 1986). The sialylation contributes to EPO's stability in the circulation (Fukuda et al., 1989).

EPO production dramatically increases in response to low partial oxygen pressure, mediated by the activation of hypoxia-inducible factor (Franke et al., 2013). In addition to hypoxia, metabolic stress, or proinflammatory cytokines can induce EPO production (Maiese et al., 2012). Upon binding to the homodimeric EPO receptor (EPOR) on erythroid progenitors in bone marrow (Marti, 2004), EPO induces JAK2 phosphorylation and the activation of downstream signaling pathways, including STAT5, PI3K/Akt, and MAPK, which enhance the proliferation, differentiation, and survival of the progenitors, thereby increasing the number of circulating erythrocytes (Quelle et al., 1996; Zhao et al., 2006). Cloned in 1985 (Jacobs et al., 1985; Lin et al., 1985), recombinant human EPO (rhEPO) has been used as a treatment for anemia (mainly caused by chronic renal failure) for more than 20 years.

### EPO and EPOR in the CNS

After EPO's hematopoietic functions were reported, subsequent studies have indicated that EPO is produced in other adult organs in addition to the kidney, such as the liver, spleen, and CNS, in response to hypoxia (Marti, 2004; Chateauvieux et al., 2011; Lombardero et al., 2011). The expression of EPO and EPOR in the brain is reported in rodents, monkeys, and humans during development and in adulthood (Marti et al., 1996; Juul et al., 1998; Knabe et al., 2004), and especially in the periventricular germinal zone of the fetal brain (Liu et al., 1997; Juul et al., 1999; Tsai et al., 2006). Although their expression decreases dramatically during the course of development, EPO and EPOR continue to be expressed in the adult V-SVZ (Liu et al., 1997).

Astrocytes were the first cells to be identified as EPO producers in the brain (Masuda et al., 1994; Marti et al., 1996). Later, neurons in several regions were also found to produce EPO (Bernaudin et al., 1999, 2000; Siren et al., 2001). Although much less EPO is produced in the brain than in the kidney (Tan et al., 1992), the locally produced EPO might play an important role in the brain, because EPO in the periphery cannot efficiently cross the blood-brain barrier (BBB) via lipid-mediated transport under normal conditions, due to its large molecular size.

Intriguingly, compared to the EPO in the circulation, brain-derived EPO has less sialylation, and consequently exhibits a smaller molecular weight and shorter plasma half-life, but it has a higher affinity for EPOR (Masuda et al., 1994). EPO production in the brain is induced by hypoxia, although the time course is quite different from that in the periphery. During hypoxia exposure, the brain EPO mRNA level rises rapidly, and this increased level is sustained for more than 24 h, whereas the circulating EPO protein and EPO mRNA in the kidney quickly decline to basal levels even under conditions of continuous hypoxia (Chikuma et al., 2000). Taken together, these findings indicate that EPO in the brain has a distinct bioactivity and regulatory system from that in the circulation.

EPOR is also expressed in various cell types in the brain, including neurons, astrocytes, OPCs, microglia, and endothelial cells (Brines et al., 2000; Nagai et al., 2001; Sugawa et al., 2002; Marti, 2004). EPOR's expression is induced by hypoxia (Chin et al., 2000; Yu et al., 2002), proinflammatory cytokines (Nagai et al., 2001), and EPO (Chin et al., 2000), and its distribution corresponds to that of EPO, suggesting that brain EPO works in a paracrine/autocrine manner in response to hypoxia. In neuronal cells, in addition to the STAT5, PI3K/Akt, and MAPK pathways, NF-κ B is involved in EPO-EPOR signaling (Digicaylioglu and Lipton, 2001; Yu et al., 2002).

### Function of endogenous EPO signaling in the brain

While a lack of EPO-EPOR signaling causes embryonic lethality with severe anemia (Wu et al., 1995; Lin et al., 1996), the brain-specific knockout of EPOR from the late embryonic phase (using EPOR-floxed mice crossed with human GFAP-Cre mice) and selective EPOR knock-in in the hematopoietic tissues of EPOR-null mice cause only a small defect in brain development (Suzuki et al., 2002; Tsai et al., 2006; Chen et al., 2007), suggesting that EPO signaling is not deeply associated with brain development. However, interestingly, EPOR is expressed in nestin-expressing NSCs in the ganglionic eminence, including in the area that develops into the postnatal V-SVZ, and hypoxia-induced EPO expression in NSCs *in vitro* promotes neuronal differentiation (Shingo et al., 2001). EPOR is also expressed in EGFR-expressing neuronal progenitors (mostly transit-amplifying progenitors) in the adult V-SVZ. In an ischemic stroke model, brain-specific EPOR knockdown did not affect the infarct volume, but it suppressed reactive cell proliferation in the V-SVZ and the migration of neuroblasts to the injury site (Tsai et al., 2006). These findings indicated that endogenous EPO-EPOR signaling in the brain is involved in controlling neurogenesis under physiological and pathological conditions.

## CNS protection by EPO treatment

### Protective effects of EPO on neurons and oligodendrocytes

A number of studies have revealed beneficial effects of EPO administration in various animal models for CNS diseases (Ghezzi and Brines, 2004; Maiese et al., 2004; van der Kooij et al., 2008) and in patients with ischemic stroke, schizophrenia, and multiple sclerosis (Siren et al., 2009). Recombinant human EPO (rhEPO) treatment directly protects neurons from hypoxia, excitotoxins, and metabolic stresses *in vitro* through multiple pathways, such as by blocking calcium influx-induced glutamate release, enhancing anti-apoptotic and anti-oxidant protein production, and suppressing pro-apoptotic protein and free radial production (Ghezzi and Brines, 2004; Marti, 2004; van der Kooij et al., 2008; Maiese et al., 2012). The peripheral administration or intraventricular infusion of rhEPO protects neurons in a number of experimental disease models, including those for ischemic stroke, traumatic injury, neurodegenerative diseases, seizure, and schizophrenia, by modifying the immune reaction and degree of inflammation, protecting the BBB, promoting angiogenesis to restore the oxygen supply, and suppressing brain atrophy and secondary gliosis, in addition to EPO's direct neuroprotective activities (Ghezzi and Brines, 2004; Maiese et al., 2004; Noguchi et al., 2007; van der Kooij et al., 2008; Chateauvieux et al., 2011).

rhEPO treatment also protects oligodendrocytes and OPCs and prevents demyelination in animal models of multiple sclerosis, spinal cord injury, and stroke (Zhang et al., 2005; Savino et al., 2006; Vitellaro-Zuccarello et al., 2007; Mizuno et al., 2008). While several studies support the finding that EPOR is expressed on OPCs (Nagai et al., 2001; Sugawa et al., 2002; Kato et al., 2011), whether or not it is expressed on mature oligodendrocytes is still controversial. It is possible that EPO stimulates other EPOR-expressing cell type(s), thereby indirectly promoting the survival of oligodendrocytes.

With regard to how peripherally administered EPO reaches the brain, both receptor-mediated active transport and extracellular pathways are used to pass EPO through the BBB (Brines et al., 2000; Banks et al., 2004; Ehrenreich et al., 2004; Juul et al., 2004; Xenocostas et al., 2005). After a single high-dose intravenous injection, EPO is detectable in the brain within hours, reaching a peak concentration at 3–4 h in the brain or cerebrospinal fluid in humans and other animals (Banks et al., 2004; Ehrenreich et al., 2004; Juul et al., 2004; Xenocostas et al., 2005). Although the influx of EPO is restricted in the healthy brain, the permeability of the BBB is significantly increased by brain insults such as ischemic stroke (Yang and Rosenberg, 2011), which can increase the ability of peripherally administered EPO to cross into the brain.

### Receptors for EPO in cytoprotection

In addition to the homodimeric EPOR, EPO binds to a heterodimeric receptor consisting of the classical EPOR and the beta common receptor (βcR), a subunit also known as CD131 and shared by several cytokine receptors, including those for interleukin (IL)-3, IL-5, and granulocyte-macrophage colony stimulating factor (Hanazono et al., 1995; Jubinsky et al., 1997). While the hematopoietic activity of EPO depends on the homodimeric EPOR, other effects appear to be mediated by the heterodimeric receptor, which has a lower affinity for EPO. For example, EPO does not exert its protective effect on spinal cord injury in mice lacking βcR (Brines et al., 2004). Moreover, EPO derivatives that do not bind the homodimeric EPOR can still protect tissue and improve neurological function similarly to EPO in stroke, spinal cord injury, and demyelination models (Brines et al., 2004; Leist et al., 2004; Savino et al., 2006; King et al., 2007; Villa et al., 2007; Wang et al., 2007b), indicating that the tissue-protection function of EPO might be mediated by the βcR-containing heterodimeric receptor.

On the other hand, the expression level of βcR in the brain is relatively quite low, and its localization does not correspond to the expression of EPO/EPOR or to the cell types protected by EPO treatment in culture or in a pilocarpine-induced epilepsy model (Nadam et al., 2007; Um et al., 2007; Sanchez et al., 2009). EPO inhibits apoptosis in neuron-like cell lines in which the βcR expression is undetectable, and the interaction between EPO and homodimeric EPOR appears to be essential for this effect (Um et al., 2007). These data suggest that EPO needs to interact with classical homodimeric EPOR to exert its neuroprotective effect. However, the functional difference between the homodimeric and heterodimeric EPOR in the V-SVZ has not been demonstrated.

### Derivatives of EPO used for cytoprotective treatment

Many derivatives of EPO have a cytoprotective effect similar to that of EPO itself (Jerndal et al., 2010). Daberpoetin-alpha, an EPO derivative with 3 times the circulation half-life of endogenous EPO, due to having more sialic acid moieties, is not only beneficial for treating anemia, but also has a neuroprotective activity similar to that of EPO in stroke, intracerebral hemorrhage, and acute ethanol intoxication models (Seymen et al., 2013; Belayev et al., 2005; Grasso et al., 2009). On the other hand, the hematopoietic activity of EPO and its derivatives sometimes causes polycythemia and thrombosis, making them inappropriate for clinical use to treat CNS disorders. Therefore, the effect of derivatives with reduced or no hematopoietic activity has been investigated in several disease models (Leist et al., 2004; Villa et al., 2007).

Asialo erythropoietin (AEPO), which is generated by the total enzymatic desialylation of rhEPO, binds to the homodimeric EPOR with a similar affinity as EPO, but is rapidly cleared from the circulation by hepatic cells, due to the lack of sialic acid at the terminals of its oligosaccharides (plasma half life, EPO: 5.6 h, AEPO: 1.4 min by intravenous administration) (Fukuda et al., 1989; Imai et al., 1990). Notably, *in vitro* experiments revealed that, while the induction of hematopoiesis requires EPO stimulation of long duration, only 5 min of EPO exposure is sufficient for neuroprotection (Morishita et al., 1997). We and others further revealed that repeated AEPO administration biweekly for more than 1 month in adult mice (Erbayraktar et al., 2003) or once a day for 2 consecutive weeks in neonatal mice (Kako et al., 2012) did not enhance erythropoiesis. Nevertheless, AEPO protects neurons (Erbayraktar et al., 2003; Wang et al., 2004b; Grasso et al., 2006) and oligodendrocytes (Savino et al., 2006) in stroke, spinal cord injury, sciatic nerve injury, and multiple sclerosis models. Since AEPO is a natural physiological metabolite of EPO, it appears to be a promising and safe drug for clinical applications.

Carbamylated erythropoietin (CEPO) is a derivative produced by chemically replacing all the lysine residues in EPO with homocitruline, a process called carbamylation. CEPO has a similar plasma half-life as EPO. Although CEPO does not bind to homodimeric EPOR, which is responsible for EPO-induced hematopoiesis (Leist et al., 2004), CEPO treatment protects neurons and oligodendrocytes from apoptosis, and it protects tissue and suppresses inflammation in brain and spinal cord injury models and cultures, as effectively as EPO (Brines et al., 2004; Leist et al., 2004; Montero et al., 2007; Villa et al., 2007; Wang et al., 2007b; Liu et al., 2011; Xiong et al., 2011). CEPO can bind to the EPOR and βcR heterodimeric receptor, and this binding was reported to mediate CEPO's cytoprotective effect in spinal cord injury (Brines et al., 2004). However, because CEPO is not a natural metabolite, careful investigation is needed to evaluate its safety for clinical applications.

## Promotion of neurogenesis/oligodendrogenesis in the V-SVZ by EPO treatment

While most previous studies suggest that EPO and its derivatives should be administered before or immediately after injury to elicit their tissue-protective effects in various animal models (van der Kooij et al., 2008), delaying administration for 24 h or even for several days after the insult can enhance neurogenesis and/or oligodendrogenesis, and lead to improved neurological symptoms weeks to months after the insult (Wang et al., 2004a; Iwai et al., 2010; Zhang et al., 2010; Kako et al., 2012) (Table 1). Although the functional aspects of the new neurons/oligodendrocytes are still unclear, infusion of an anti-mitotic agent, Ara-C, which inhibits neurogenesis, effectively abolished the functional recovery after EPO treatment in a traumatic injury model (Zhang et al., 2012), suggesting that the EPO-induced functional improvement depends, at least in part, on the production of new neurons/oligodendrocytes after the injury.

**Table 1:** **EPO's effects on V-SVZ neurogenesis/oligodendrogenesis**.

**Model**	**Age**	**EPO treatment**	**Effects on neurogenesis, oligodendrogenesis, and angiogenesis**	**References**
Rat, stroke	Adult	rhEPO, i.p., 1-8 dpi	V-SVZ: increase in NSCs and neuroblasts St, Cx: increase in neuroblasts and blood vessels	Wang et al., 2004a, 2006a
Rat, stroke	Adult	rhEPO, i.p., 0-4 dpi	V-SVZ: increase in cell proliferation and OPCs Cx: increase in angiogenesis	Kim and Jung, 2010
Rat, stroke	Adult	rhEPO, i.p., 1-8 dpi	(Lentivirus injection into V-SVZ 3 d before injury) V-SVZ: increase in OPCs and OPC proliferation St: increase in virus-labeled OPCs	Zhang et al., 2010
Mouse, stroke	Adult	rhEPO, with hydrogel, epicortical, 4 or 11 dpi	V-SVZ: increase in neuroblasts St, Cx: increase in neuroblasts	Wang et al., 2012
Rat, stroke	Neonate (P 10)	rhEPO, i.p. 0 dpi	St: increase in mature new neurons	Gonzalez et al., 2007
Rat, stroke	Neonate (P 7)	rhEPO, i.p. 0, 1, 7 dpi	(Lentivirus injection into LV at P1) St, Cx: increase in virus-labeled neuroblasts and OPCs, decrease in virus-labeled astrocytes	Gonzalez et al., 2013
Rat, H/I	Neonate (P 7)	rhEPO, i.p. 0, 2, 4, 6 dpi	V-SVZ: increase in proliferating cells and neuroblasts St, Cx: increase in neuroblasts, mature new neurons, and blood vessels	Iwai et al., 2007
Rat, H/I	Neonate (P 7)	rhEPO, i.p. 2, 4, 6, 9, 13 dpi	St, Cx: increase in neuroblasts St, Cx, CC: increase in OPCs and oligodendrocytes Cx, CC: increase in BrdU-labeled oligodendrocytes	Iwai et al., 2010
Mouse, H/I	Neonate (P 9)	rhEPO, i.p. 0, 1, 2 dpi	V-SVZ: increase in proliferating cells (only in females)	Fan et al., 2011
Mouse, H/I	Neonate (P 5)	AEPO, i.p.,5-19 dpi	(Retrovirus injection into V-SVZ at P5) CC: promotion of oligodendrocyte differentiation	Kako et al., 2012
Rat, 6-OHDA	Adult	rhEPO, i.c.v. for 7 d	V-SVZ: increase in neuroblast St: increase in neuroblasts	Kadota et al., 2009
Mouse, intact	Adult	rhEPO, i.c.v., for 6 d	V-SVZ: decrease in NSCs, increase in NPCs and neuroblasts	Shingo et al., 2001
Mouse, intact	Adult	EPOR-CKO	V-SVZ: decrease in cell proliferation	Tsai et al., 2006
Mouse, stroke	Adult	EPOR-CKO	V-SVZ: lack of stroke-induced increase in cell proliferation	Tsai et al., 2006
Mouse, intact	Adult	Conditionally rescued *EPOR^−/−^*	V-SVZ: decrease in cell-proliferation	Chen et al., 2007

Previous studies that report the effects of EPO treatment and EPO signaling modification on V-SVZ-associated neurogenesis and oligodendrogenesis. i.c.v., intracerebroventricular; i.p., intraperitoneal; dpi, days post injury; St, striatum; Cx, cortex; P, postnatal day; H/I, hypoxia/ischemia; CKO, conditional knockout.

### Promotion of neurogenesis by EPO treatment

rhEPO administration promotes neurogenesis under both physiological and pathological conditions. In the intact brain, rhEPO infusion for 6 days into the lateral ventricle increases the number of neuronal progenitor cells, with a concomitant decrease in the number of NSCs, suggesting that EPO enhances neuronal differentiation (Shingo et al., 2001). Furthermore, in stroke and neonatal hypoxia/ischemia models, peripherally administered rhEPO increases the numbers of both NSCs and neuronal progenitors by promoting their proliferation and neuronal differentiation, and it enhances the migration of neuroblasts to the injury site (Wang et al., 2004a; Iwai et al., 2007). The intraventricular infusion of rhEPO in 6-OH-DOPA-injected Parkinson's disease model animals also enhances neuroblast production in the V-SVZ and increases the number of neuroblasts within the striatum (Kadota et al., 2009).

With regard to how EPO affects neurogenesis, *in vitro* studies suggest that it has direct effects on NSCs. For example, in NSCs dissociated from the adult V-SVZ, EPO treatment increases Akt activity, which promotes proliferation, differentiation, and neurite outgrowth; neurogenin-1 is involved in this pathway (Wang et al., 2006b). In a similar culture system, CEPO activates sonic hedgehog to induce the expression of Mash1, a bHLH transcription factor that increases neurogenesis (Wang et al., 2007a). In embryonic NSCs, EPO enhances the nuclear translocation of NF-κB to promote neuronal differentiation and induces Mash1 expression (Shingo et al., 2001).

EPO can also enhance neurogenesis through the protection and remodeling of the cerebral vasculature. As previously mentioned, the vasculature is involved in maintaining the function of NSCs and transit-amplifying neuronal progenitors in the V-SVZ. In addition, after ischemia, endothelial cells secrete attractive molecules such as SDF-1 and angiopoietin-1, which guide neuroblasts expressing receptors for these molecules toward the injury (Imitola et al., 2004; Ohab et al., 2006; Robin et al., 2006). Blood vessels also appear to act as a scaffold for the migrating neuroblasts in the striatum after stroke (Yamashita et al., 2006; Kojima et al., 2010) and for V-SVZ-derived progenitors that give rise to oligodendrocytes in the demyelinated corpus callosum (Cayre et al., 2013). EPO treatment suppresses the apoptosis of vascular endothelial cells in which EPOR expression is strongly induced by ischemia (Bernaudin et al., 1999; Chong et al., 2002), and promotes angiogenesis to restore blood flow in the ischemic area (Li et al., 2007). This angiogenesis is reported to be mediated by the enhanced endothelial expression of VEGF, a critical growth factor for generating and remodeling the vasculature (Wang et al., 2004a). EPO also induces matrix metalloproteinase (MMP)-2 and MMP-9 in endothelial cells, via activation of the Akt and ERK1/2 pathway, to promote neuroblast migration (Wang et al., 2006a). Interestingly, EPO-treated neurospheres derived from the adult V-SVZ promote the capillary-like tube formation of cultured endothelial cells by secreting VEGF (Wang et al., 2008). Therefore, EPO's efficient enhancement of neurogenesis and oligodendrogenesis appears to be tightly associated with vascular function.

### Promotion of oligodendrogenesis by EPO treatment

In addition to preventing myelin degeneration (Zhang et al., 2005; Savino et al., 2006; Vitellaro-Zuccarello et al., 2007; Liu et al., 2011), several studies have reported that delaying EPO treatment by 1–7 days after the insult, which doesn't protect the tissue from injury, efficiently enhances the production of new oligodendrocytes (Iwai et al., 2010; Zhang et al., 2010; Kako et al., 2012) and promotes white matter reorganization (Li et al., 2009) leading to improved neurological function. While the underlying mechanism remains unknown, these observations suggest that EPO treatment promotes remyelination to alleviate neurological dysfunction.

Since oligodendrogenesis takes place not only in localized areas, but also widely throughout the brain even in adulthood, especially after demyelination, a region-specific cellular labeling method is needed to determine the effect of EPO treatment on the behavior of V-SVZ-derived OPCs/oligodendrocytes. For this purpose, viral vectors were stereotaxically injected into the rodent V-SVZ (Zhang et al., 2010; Kako et al., 2012). The lentivirus vector is efficiently integrated into the genome of both proliferating and non-proliferating cells, permanently labeling them and their progenies. Using this system, a 7-day treatment with EPO starting 24 h after ischemic stroke was shown to increase the OPCs in the V-SVZ, and to promote the recruitment to the injury site of lentivirus-labeled cells that express a mature oligodendrocyte marker (Zhang et al., 2010). In addition, in a neonatal stroke model, EPO treatment was shown to promote the proliferation of lentivirus-labeled NSCs, which preferentially produced neurons and oligodendrocytes rather than astrocytes in the injured striatum (Gonzalez et al., 2013).

To investigate the specific effect of EPO treatment on V-SVZ-derived OPCs, we combined a retrovirus injection into the V-SVZ (which labels proliferating cells and their progenies) with a lineage-specific fate-mapping method for OPCs, using tamoxifen-induced recombination, in a neonatal ischemic injury model (Kako et al., 2012). Even though V-SVZ-derived OPCs showed extensive migration in the corpus callosum toward the injured white matter, only about 30% of them underwent maturation in the subsequent 19 days. The maturation ratio was significantly lower than that in the intact brain, in which more than 60% of OPCs differentiated into mature oligodendrocytes over the same time period. Interestingly, this maturation defect in the injured area was relatively mild, when we considered the entire OPC population, in which parenchymal OPCs predominate and the percentage of OPCs derived from the V-SVZ is small. These data suggest that V-SVZ-derived OPCs/oligodendrocytes are more susceptible to an inflammatory environment than are parenchymal OPCs. Delayed AEPO treatment given for a duration of 2 weeks, but not for 3 days, could almost completely prevent this maturation defect and neurological impairment. Taken together, these findings indicate that EPO treatment enhances OPC production in the V-SVZ and promotes the migration and differentiation of V-SVZ-derived OPCs to supply new oligodendrocytes in the injured brain.

**Figure 1:**
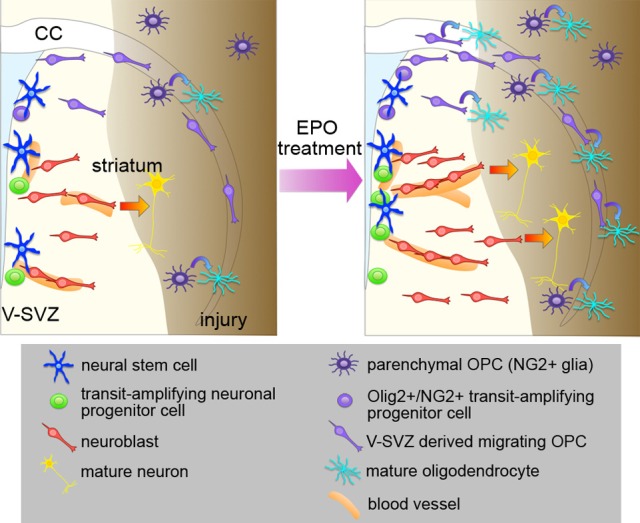
**Effects of EPO treatment on the V-SVZ after brain injury**. Schematic representation of the effects of EPO treatment on neurogenesis, oligodendrogenesis, and angiogenesis in the brain after injury. EPO treatment enhances the proliferation of NSCs and neuronal progenitors to increase the number of neuroblasts after injury, and promotes recruitment of the neuroblasts to the injured area, where they differentiate into mature neurons. EPO treatment increases the production of migrating OPCs from the Olig2+/NG2+ transit-amplifying progenitors in the V-SVZ, and promotes the differentiation of V-SVZ-derived OPCs into oligodendrocytes in the injured area. The proliferation and differentiation of the parenchymal OPCs are also enhanced by EPO. EPO also enhances angiogenesis, which might be involved in the EPO-induced neurogenesis and oligodendrogenesis after injury.

In summary, many studies support the idea that EPO enhances neurogenesis and/or oligodendrogenesis at multiple steps after brain insults (Figure 1). However, in most of these studies, EPO was administered using the same protocols as those of the previous studies that demonstrated EPO-induced neuroprotection; thus, the optimal dosage, timing, and duration of EPO treatment for promoting regeneration have not been carefully investigated. Considering that regeneration is a continuous process occurring over months to years, which is much longer than the process of cell death, which mostly takes place during the acute phase after insult, the optimal EPO treatment schedule for efficient regeneration should be different from that used for cytoprotection.

## Perspectives

EPO and its derivatives appear to be promising drugs for promoting neuron and oligodendrocyte regeneration by V-SVZ-derived progenitors in various CNS disorders, as discussed above. However, fundamental questions remain to be addressed. For example, it is not known whether the V-SVZ-derived oligodendrocytes induced by the EPO treatment are functionally similar to those derived from parenchymal OPCs. In addition, the effect of EPO on neurogenesis and oligodendrogenesis in the primate V-SVZ remains to be studied. Furthermore, the underlying mechanisms of EPO's effects are still largely unknown, especially those responsible for EPO's effect on V-SVZ oligodendrogenesis.

Notably, systemic rhEPO treatment has several potential risks, including not only polycythemia and thrombosis, due to its hematopoietic activity, but also hypertension due to increased vascular smooth muscle contraction (Vaziri et al., 1995; Miyashita et al., 2004) and the promotion of malignant tumor growth by enhanced tumor cell survival and angiogenesis (Yasuda et al., 2003). Therefore, further studies to determine the mechanisms of each effect of EPO and its derivatives should help guide the development of appropriate treatments that specifically promote neurogenesis and oligodendrogenesis without causing adverse events.

## Author contributions

Naoko Kaneko: conception and design, collection and assembly of data, and manuscript writing, Eisuke Kako: collection and assembly of data, Kazunobu Sawamoto: conception and design, manuscript writing, and financial support

### Conflict of interest statement

The authors declare that the research was conducted in the absence of any commercial or financial relationships that could be construed as a potential conflict of interest.
